# An Integrative Theory-Driven Positive Emotion Regulation Intervention

**DOI:** 10.1371/journal.pone.0095677

**Published:** 2014-04-23

**Authors:** Fanny Weytens, Olivier Luminet, Lesley L. Verhofstadt, Moïra Mikolajczak

**Affiliations:** 1 Research Institute for Psychological Sciences, Université catholique de Louvain, Louvain-la-Neuve, Belgium; 2 National Fund for Scientific Research (FRS-FNRS), Brussels, Belgium; 3 Department of Experimental Clinical and Health Psychology, Ghent University, Ghent, Belgium; University of Missouri-Kansas City, United States of America

## Abstract

Over the past fifteen years, positive psychology research has validated a set of happiness enhancing techniques. These techniques are relatively simple exercises that allow happiness seekers to mimic thoughts and behavior of naturally happy people, in order to increase their level of well-being. Because research has shown that the joint use of these exercises increases their effects, practitioners who want to help happiness seekers need validated interventions that combine several of these techniques. To meet this need, we have developed and tested an integrative intervention (Positive Emotion Regulation program – PER program) incorporating a number of validated techniques structured around a theoretical model: the Process Model of Positive Emotion Regulation. To test the effectiveness of this program and to identify its added value relative to existing interventions, 113 undergraduate students were randomly assigned to a 6-week positive emotion regulation pilot program, a loving-kindness meditation training program, or a wait-list control group. Results indicate that fewer participants dropped out from the PER program than from the Loving-Kindness Meditation training. Furthermore, subjects in the PER group showed a significant increase in subjective well-being and life satisfaction and a significant decrease in depression and physical symptoms when compared to controls. Our results suggest that the Process Model of Positive Emotion Regulation can be an effective option to organize and deliver positive integrative interventions.

## Introduction

Until recently, research has focused on factors that hinder well-being, with the objective of identifying means by which to ease suffering [1]. Over the last fifteen years, however, many psychology researchers have insisted on the necessity to extend these studies by analyzing the individual differences and the processes that contribute to well-being [2]. One objective of this latter research is to highlight individuals' available means to boost their subjective happiness. According to Duckworth, Steen, and Seligman [3], these means are threefold: increasing pleasure, boosting engagement, and finding meaning in life. As pleasure is the fruit of experiencing positive emotions, increasing their frequency, intensity, or duration is one of the avenues through which individuals can achieve higher levels of happiness [4]. Indeed, a growing number of cross-sectional, longitudinal, and experimental studies show that positive emotions play a key role in individuals' evaluation of their level of well-being and contribute to numerous related benefits (for a review, see [5]). For example, at the cognitive level, experimental studies have shown that inducing positive emotions broadens individuals' scope of attention [6] and increases their creativity [7]. At the somatic level, longitudinal studies have shown that positive emotions are associated with increased longevity [5], [8]–[11], which is not surprising, as experience of positive affect was associated with better immunity in cross-sectional [12], [13] and experimental studies [14], [15]. At the social level, positive affect is related to better interpersonal relationships [16]–[20] and generally increases altruism [21], [22]. As a whole, these studies point to the importance of positive affect at both the individual and the societal levels and highlight the importance of developing interventions aimed at increasing positive affect and, consequently, well-being.

To provide clinicians with practical and efficient interventions, researchers have created and validated a set of techniques that enable individuals to increase their positive emotions and their levels of well-being (for a review, see [23]). In this area of research, techniques were frequently tested one at a time and presented to participants as one-shot exercises. This way of proceeding enables researchers to test the efficacy of each exercise individually. However, recent research [24] has shown that this way of testing interventions has low ecological validity because, in naturalistic settings, happiness seekers often practice (and wish to practice) multiple exercises at the same time. Furthermore, the use of multiple techniques in an intervention often produces better results than when a single technique is used alone [24].

Several authors have tested interventions aimed at consolidating multiple techniques, provided as either individual or group sessions (e.g. [25]–[27]). *Criteria* used to determine a technique's inclusion in those programs were not always clearly defined or explicitly reported. In the 1980s, Fordyce [25], [26] gathered a set of *exercises aimed at teaching happiness seekers to mimic typical behaviors and thinking styles of happy people*. The exercises included in Fordyce's program encouraged participants to: (a) keep busy and be more active; (b) spend more time socializing; (c) be productive at meaningful work; (d) get better organized and plan things out; (e) stop worrying; (f) lower expectations and aspirations; (g) develop positive, optimistic thinking; (h) become present-oriented; (i) work on a healthy personality; (j) develop an outgoing, social personality; (k) be yourself; (1) eliminate negative feelings and problems; (m) see close relationships as the number one source of happiness; (n) put happiness as your most important priority ([26], p.484). In a more recent attempt, Seligman, Rashid, and Parks [27] created a program gathering the *“best-documented exercises” in the literature* ([27], p. 776) *targeting at least one of the three components of “happiness,”* as defined by Seligman (i.e., positive emotions, engagement, and meaning) [4]. Exercises integrated in Group Positive Psychotherapy were the following: (a) using your strengths; (b) three good things/blessings; (c) obituary/biography; (d) gratitude visit; (e) active/constructive responding; and (f) savoring ([27], p. 776).

Although these studies provided essential information about the potential of integrative interventions, this kind of program would greatly benefit from a strong theoretical background which should (1) at a theoretical level, allow a better understanding of the mechanisms underlying happiness enhancement and (2) at a more practical level, help practitioners identify their clients' needs, weaknesses, and the most appropriate technique(s) to prescribe them [28]. As no happiness enhancing program that is both integrative and theory-driven currently exists, we decided to create such a program and to evaluate its effects on a set of mental and physical health variables. To this end, we developed an integrative program structured around a conceptual framework, which enabled us (1) to select a set of techniques involving different underlying processes and (2) to organize them in a coherent manner.

The model proposed by Quoidbach et al. [28], [29] appears to fulfill these requirements. As outlined below, Quoidbach et al. modeled positive emotion regulation strategies with reference to a well-known model in the domain of negative emotion regulation: Gross' Process Model of Emotion Regulation [30], [31]. This model enables the integration of strategies involving clearly differentiated emotion regulation processes and the reconciliation of studies on both positive and negative emotion regulation.

### The Process Model of Emotion Regulation applied to Positive Emotions

The Process Model of Emotion Regulation [30], [31] provides a theoretical structure for analyzing emotion regulation processes. The model highlights five *families* of emotion regulation strategies. Initially created to understand and organize negative emotion regulation strategies, it was later adapted by Quoidbach et al. to apply to the up-regulation of positive emotions (see The Process Model of *Positive* Emotion Regulation, Quoidbach et al. [28], [29]). The first family of strategies that can be used to influence emotion is *situation selection.* Situation selection involves “choosing or avoiding some activities, people, or places in order to regulate emotions” [32]. To illustrate this strategy, we can imagine a grandfather, Joseph, *deciding to visit* his children and grandchildren on Sunday afternoon because he knows that being in touch with his family gives him a lot of pleasure.

The second family of strategies highlighted by Gross, *situation modification,* includes techniques that allow an individual to change the situation s/he is facing (or that s/he has planned) in order to change its emotional impact. To continue our example, in order to make Sunday afternoon fun and enjoyable, Joseph may decide to bring his grandchildren’ favorite board game that they all like to play together.

*Attentional deployment* includes strategies that involve altering how an individual feels by selecting the information to which s/he attends. During Sunday afternoon, rather than getting irritated by the noise of the neighbor's lawnmower, Joseph may fully focus his attention on the fun game he is playing with his grandchildren. Along the same line, Joseph may concentrate his attention on the taste of the wonderful cheesecake his daughter prepared and deeply savor it to get the most pleasure from it.

*Cognitive change* refers to changing the way an individual thinks in order to change the way he/she feels, either by changing how he/she thinks about the situation itself or about his/her capacity to manage its demands. For instance, rather than taking the moment for granted, Joseph may interpret his presence among his family that afternoon as a gift of life.

Finally, the last family of strategies proposed by Gross is *response modulation*. This family of strategies involves techniques to alter bodily manifestations of emotion (e.g. physiological, behavioral). In order to increase his excitement and pleasure during the time he is spending with his family, Joseph may decide to smile and laugh with his loved ones and to express his affection for them.

Given that generating a high level of well-being implies reminiscing about past positive events, savoring the present, and anticipating positive future events [33], [34], [35], Quoidbach et al. [28], [29] divided Gross's model into three moments of action: before, during, and after the positive emotions generating event (for a detailed description of the model, see [28], [29]). Thus, Quoidbach et al. [28], [29] do not propose five large *families of strategies*, but rather 15 *distinct strategies* that an individual can use to regulate his/her emotions, corresponding to the 15 sections of the Process Model of Positive Emotion Regulation (see Table 1, see italics).

**Table 1:** Example of *positive emotion regulation techniques* in the Positive Emotion Regulation Model proposed by Quoidbach & al. [28], [29] for a specific positive event.

	Situation Selection (1)	Situation Modification (2)	Attentional Deployment (3)	Cognitive Change (4)	Response Modulation (5)
BEFORE (B)	*Knowing what matters*	*Creating the best possible conditions*	*Pre-experiencing*	*Optimistic outlook*	*Getting pumped-up*
	Decide to visit your grandparents who live in Quebec	Budget for the journey to plan your savings; Buy a guide and plan the trip program; Get sufficient rest before departure	Visualize the evenings to be spent together around the fireside listening to your grandparents tell family stories; Imagine the landscapes you can watch	Imagine how pleasant these moments will be	Call your grandparents to let them know how much you are looking forward to spending time with them; Count down the days to your departure on your Facebook status
DURING (D)	*Doing what matters*	*Optimizing*	*Savoring the moment*	*Positive appraisal*	*Expressing emotions*
	Actually leave despite all the “good reasons” to cancel your trip	Keep a very nice outing for the last day; Deactivate your mail box; Get up early to make the most of the time spent there	Fully immerse yourself in the moments experienced; Be receptive to the landscape's beauty	Be conscious of the chance that you have in traveling far away to meet people that you love	Celebrate the reunion; Smile; Tell your grandparents how happy you are to share those moments with them
AFTER (A)	*Remembering what matters*	*Memories crafting*	*Re-experiencing*	*Grateful outlook*	*Capitalizing*
	When you get back home, make a souvenir box with photos, scents, objects that remind you of the happy moments spent in Quebec	Throw away the bad photos; Make a scrapbooking album with photos from the trip	Replay/re-live the good moments spent there	Be conscious of your chance to have experienced such moments; Imagine how life would be without your grandparents	Visit your parents to thank them for having financed part of the journey; Tell a close friend about the trip

The use of such a framework makes it possible to classify the different types of techniques that individuals may use to maximize their positive emotions. Table 1 illustrates a positive event and the means an individual may use to increase the intensity, frequency, and duration of the positive emotions that s/he experiences. Each action (or technique) that an individual can implement in order to up-regulate his/her positive emotions with respect to a given positive event finds its place in one of the model's sections. As this model enables the integration and organization of happiness enhancing techniques, it offers an interesting framework with which to elaborate our intervention.

## The Current Study

The aim of the present study was twofold: (1) to create an integrative intervention (i.e. including multiples techniques) on the basis of a theoretical model and (2) to evaluate its impact on psychological and physical well-being variables.

### First Objective: Creation of the Positive Emotion Regulation (PER) Program

To achieve our first objective, we carried out an extensive literature review on the basis of the model proposed by Quoidbach et al. [28], [29] in order to identify a set of validated happiness enhancing techniques that we could include in our intervention. For each technique, we identified at which *moment of action* it was applicable (i.e., before, during, and/or after an event). We then evaluated the *family of strategies* to which each of these techniques corresponded. By combining this information, we were able to identify the *strategy* (or section) of the model to which each technique corresponded. This theoretical framework provided us with a structure in which to organize these validated techniques within an integrative intervention seeking to regulate positive emotions and to increase individuals' well-being.

This selection and classification process led us to include the following techniques in our intervention (see Table 2 for a detailed overview of the PER program, the extensive manual is available upon request from the first author). Techniques in the **situation selection** section aim at teaching people the kind of events they should plan (see B1 in Table 2, corresponding to “Before an event”), experience (see D1 in Table 2, “During an event”), or remember (see A1 in Table 2, “After an event”) in order to increase their level of well-being. These techniques included the following: playing sports [36], being altruistic [37], socializing [38], taking care of one's needs [39], goal setting [40], monitoring progress toward a goal [41], and identifying memories to preserve [42] or to recall [43].

**Table 2:** Week-by-Week Summary of the Positive Emotional Regulation Program.

Session 1:	
Presentation of the PER program and introduction	
Presentation of the theoretical framework	
Emotion: definition and functions	
The role of positive emotions in well-being and happiness	
Introduction to emotion regulation strategies	
Classification of positive emotion regulation strategies (Quoidbach et al.'s model, 2012)	
Happiness and positive emotions enhancing techniques	
*Techniques usable BEFORE an event*	
B1	Situation Selection: select future situations that will make you happy
1. Practice sports	
2. Be altruistic	
3. Socialize	
4. Take care of your needs	
Homework	

Session 2:	
B2	Situation Modification: modify future situations to boost happiness potential
1. Plan your events practically	
2. Optimize your events by adding pleasure	
3. Use job crafting	
4. Manage time for important domain of your life	
B3	Attentional Deployment: mentally project yourself in future pleasant events
1. List tomorrow's great things/activities	
2. Practice mental time travel about positive future events	
3. Imagine your best future self	
Homework	

Session 3:	
B4	Cognitive Change: adopt an optimistic perspective about future and reduce your expectations
1. Find positive aspects in your negative/neutral/positive future events	
2. Reduce your expectations	
B5	Response Modulation: express your emotions about future positive events
1. Diversify your means of expression	
2. Use different means to express your positive emotions for future events	
*Techniques usable DURING an event*	
D1	Situation Selection: really do what is important for your happiness, go from anticipation to action
1. Set goals	
2. Monitor your progress toward your goal	
Homework	

Session 4:	
D2	Situation Modification: modify situations to increase their positive potential
1. Live your best day	
2. Optimize the end of an event	
3. Find the flow	
D3	Attentional Deployment: fully deploy your attention to the present pleasures
1. Savor a piece of food	
2. Savor something you are doing mechanically in everyday life	
3. Be receptive to beauty or excellence	
4. Pay attention to compliments you are receiving and accept them	
D4	Cognitive Change: interpret events positively, realize your luck
1. Realize why are you responsible of your success	
2. Practice counterfactual thinking	
3. Realize your luck	
Homework	

Session 5:	
D5	Response Modulation: express your positive emotions
1. Smile	
2. Make you smile	
3. Feel positive emotions in your body and express them	
4. Show your affection	
*Techniques usable AFTER an event*	
A1	Situation Selection: identify what was important, what you should remember
1. Identify the need you took care in past positive events to know what is important to you	
2. Identify memories you should preserve	
3. Create a memorabilia box	
A2	Situation Modification: filter your memories and showcase only the good ones
1. Make a selection in your souvenirs and showcase only the good ones	
A3	Attentional Deployment: mentally relive positive past experiences
1. Practice mental time travel about past positive events	
Homework	

Session 6:	
A4	Cognitive Change: be aware of the good moments you lived
1. Keep a gratitude journal	
2. Realize what went well during the last year	
3. Write a gratitude letter	
A5	Response Modulation: express your positive emotions about past events
1. Diversify your means of expression	
2. Share past positive experiences	
3. Celebrate your and others' achievements, capitalize on them	
4. Do a gratitude visit	
Assess your progress	
Keys about sustainable happiness	
Identify exercises that worked best for you	
Plan those exercises in your everyday life	
Assessment of the training and collective debriefing	

*Note.* B  =  techniques usable Before a positive event; D  =  techniques usable During a positive event; A  =  techniques usable After a positive event. These three categories of techniques are split into five families of strategies, corresponding to Gross' Process Model of Emotion Regulation strategies: 1  =  Situation Selection; 2  =  Situation Modification; 3  =  Attentional Deployment; 4  =  Cognitive Change; 5  =  Response Modulation.

Techniques in the **situation modification** section aim to modify planned (B2) or current (D2) activities, or their memories of these activities (A2), in order to optimize their well-being potential. Techniques such as time management and planning [44], job crafting [45], optimizing the end of an event [46], finding the flow [47], and showcasing souvenirs [48] were included in this category.

**Attentional deployment** techniques help individuals to project themselves into a pleasant future (B3), to be fully present in an agreeable moment (D3), or to relive happy or meaningful moments of their lives (A3). Exercises such as imagining your best future self [49], mental time traveling to a pleasant future [50], savoring [51], and mental time traveling into past positive events [52] are examples of techniques in this category.

Techniques identified as **cognitive change** strategies aim at helping individuals to interpret future (B4), current (D4), and past events (A4) in a positive way and to make individuals aware of the good things in their lives. This category of techniques includes optimistic thinking [53], reducing one's expectations [54], counting one's blessings [55], counterfactual thinking [56], writing a gratitude letter [57], and what-went-well exercises [58].

Finally, in the **response modulation** category, we gathered techniques used to facilitate positive emotion expression for future (B1), current (D5), and past events (A5). Included exercises are sharing excitement for upcoming events [59], smiling [60], sharing good news with others [61], capitalizing [62], and gratitude visits [63].

### Second Objective: Evaluation of the PER Program's Effectiveness

Our second objective was to quantify the effectiveness of the developed program. To achieve this, we sought to compare the results of the participants in the PER program to those of individuals who didn’t participate in any intervention program (control group on a wait list). To identify the potential added value of our PER program to existing methods, we sought to compare the PER results to those of an intervention that (1) also seeks to increase positive emotions and well-being, (2) has been previously validated, and (3) can be administered in six two- to three-hour weekly sessions, followed by exercises to be completed at home. A literature review enabled us to identify the loving-kindness meditation (LKM), “a meditation technique used to increase feelings of warmth and caring for self and others” [64], as a technique that fulfilled all these criteria. Fredrickson, Cohn, Coffey, Pek, and Finkel [65] have shown that practicing this type of meditation significantly increases positive emotions as well as life satisfaction, while decreasing depression symptoms. The other types of validated interventions proved unsatisfactory as a control group, as it seemed inappropriate, and possibly counter productive, to organize an intervention around, for instance, an exercise such as “Counting One's Blessings” (e.g. [55]) and to ask participants to practice only this exercise during the six weeks of intervention (two hours/week in a group session, in addition to the follow-up exercises to be done at home). As our objective was to compare two interventions with a common aim, similar pace, and equivalent duration, LKM seemed to be the most appropriate validated option to serve as a point of comparison to adequately measure the effectiveness of the PER program.

Thus, by comparing these three conditions (PER program, LKM, and the control) we tested the hypothesis that, compared to the control group, the two interventions would *enhance subjective happiness and life satisfaction and diminish depression symptoms, the frequency of physical symptoms, as well as perceived stress, as* measured through self-report questionnaires.

### Methods

#### Ethics Statement

This study was approved by the Ethics Committee of the Psychology Department at the Université catholique de Louvain, Belgium, and was conducted in accordance with the Declaration of Helsinki.

#### Participants and Procedure

One hundred and thirteen undergraduate students (88 women; mean age  =  22.29, SD  =  2.49) took part in the study on a voluntary basis without any financial or course credit compensation (in order to guarantee intrinsic motivation to participate). They responded to an advertisement referring to “a six-week happiness enhancing training”. After providing written informed consent, they were randomly assigned to one of the three groups: the Positive Emotion Regulation (PER) intervention (N = 36; 32 women), the Loving-Kindness Meditation (LKM) intervention (N = 35; 27 women), or the waiting list (control group; N = 42; 29 women). The study advertisement was intentionally vague to keep participants blind to the multiple conditions and to allow a random assignment between the three.

Measures were taken one week before the intervention (T1) and four weeks after (T2), to evaluate medium term results. Among the participants who followed the complete 6-week training program (PER, N = 32; LKM, N = 20, attrition discussed below), four in the PER condition and four in the LKM condition failed to complete T2 measurements; they were, therefore, removed from the sample. In the control condition, seven participants did not complete the T2 assessment; they were also excluded from analyses. Analyses of mental health and physical variables presented below are, therefore, based on a final sample of 79 participants broken down as follows: PER, N = 28 (24 women, mean age = 22.5, SD = 3.06); LKM, N = 16 (13 women, mean age = 22.25, SD = 1.7); and control group, N = 35 (24 women, mean age = 22.14, SD = 2.35). Note that there were no significant differences on baseline measures between participants who completed T2 measures and those who did not (ps ranging from.086 to.897).

#### Trainings format

The Positive Emotion Regulation (PER) and the Loving-Kindness Meditation (LKM) interventions were developed according to the same format (length, pace, and organization) and differed only in terms of content. The participants of each of these two conditions were divided into three small groups comprising ten to 14 individuals in order to establish effective group dynamic conducive to learning. For six consecutive weeks, both experimental groups participated in once-weekly, two-hour training sessions. LKM and PER group sessions sought to give participants a theoretical framework to understand the importance of the presented techniques on the one hand and, on the other, to enable them to experiment with and to practice certain exercises during the session before implementing them at home. Our program was therefore structured around theoretical and experiential methods [66] in order to maximize learning transfer (see [67], [68] for reviews and transfer guidelines).

#### Conditions

*Loving-kindness meditation:* This training was a French adaptation of the training proposed by Fredrickson et al. [65] and was created on the basis of the material we received from S. Finkel, the LKM trainer in this study. The only difference was in relation to the duration of the “on-site” sessions that were two hours long (as opposed to 60 minutes in the session proposed by Fredrickson et al. [65]), so that the duration of the LKM intervention could be comparable to that of the PER program. As Sin and Lyubomirsky [23] have shown that the duration of an intervention using happiness enhancing techniques is positively correlated to its effectiveness, we considered this modification to be essential. The longer session duration was achieved by adding basic meditation exercises as well as by increasing the duration of the “on-site” LKM exercises and of their debriefing. As in the Fredrickson et al. study [65], the LKM group participants were asked to practice 20 minutes of meditation at home, at least five days a week. Participants were strongly encouraged to continue their LKM practice after the end of the program. LKM sessions were led by two highly experienced mindfulness trainers (Christophe Dierickx and Gwenaëlle Rivez) that were trained in LKM through multiple readings, significant amounts of practice, and supervisions.

*Positive Emotion Regulation:* As we explained in the introduction, the intervention was structured around the Process Model of Positive Emotion Regulation. During the group sessions, each section of the model was set out and put into practice through validated techniques (see Table 2 for examples). Research findings were presented to highlight the pertinence of the strategies and techniques proposed. The presentation of the strategies relative to the different sections of the models was divided across six sessions. As in the LKM condition, PER group participants took part in the exercises proposed during the session and received a list of exercises to carry out at home for the following week (combination of mandatory and self-selected exercises for a duration equivalent to 5 × 20 minutes). At the end of the program, participants were asked to identify exercises that worked the best for them (which is not necessary equal to their preferred ones, [69], see discussion section for more details), and were strongly encouraged to continue them after the end of the intervention. PER trainers (five psychology students and one Ph.D. student, operating in pairs) were a little less experienced group trainers than LKM trainers (which is why they worked in pairs), but were highly knowledgeable about positive emotion regulation topics.

#### Measures

*Subjective Happiness* was measured using the well-validated Subjective Happiness Scale (SHS; [70]). This questionnaire provides a global, subjective assessment of whether the respondent considers himself/herself as a happy or an unhappy person, via four items rated on a 7-point Likert scale. The internal consistency (α) was.91 at T1 and.87 at T2.

*Life Satisfaction* was assessed with the Satisfaction with Life Scale (SWLS; [71]). This scale comprises five items (e.g. “So far I have gotten the important things I want in life”) rated on a 7-point scale. The internal consistency was.81 at T1 and.86 at T2.

*Depression* was measured via the Beck Depression Inventory (BDI; [72]). In the present study, we used the short version [73], which consists of 13 items rated on a 4-point scale. Respondents were instructed to choose the response that best described how they felt over the last week. The internal consistency of the scale was.82 at T1 and.87 at T2.

*Perceived Stress* was evaluated using the Perceived Stress Scale (PSS; [74]). This scale comprises 10 items (e.g. “In the last month, how often have you felt that you were unable to control the important things in your life?”) rated on a 5-point scale (0 = never, 1 = almost never, 2 = sometimes, 3 = fairly often, 4 = very often). The internal consistency was.86 at T1 and.69 at T2.

*Somatic complaints* were assessed through a short version of the Pennebaker Inventory of Limbic Languidness (PILL; [75]; short-version [76]). The abbreviated scale consists of a list of the 29 most common physical symptoms (e.g., headache, stomachache, sleep problems, cramps). Participants had to rate on a 5-point scale the frequency with which they experienced each symptoms/sensation during the past month (1  =  never or nearly never, 2 = 1 to 3 times, 3 = every week, 4 = several times a week, 5 = every day). The internal consistency was.86 at T1 and.91 at T2.

### Results

#### Preliminary results

Dropout analysis revealed that among the 36 participants assigned to the PER group, four of them left the program before its end (11% of the subjects). Among the 35 participants assigned to the LKM condition, 15 of them withdrew before the end ( =  43%). A Chi-square test revealed that the dropout difference between the two groups was significant, X^2^ (1, *N* = 71) = 9.125; p<0.003. No differences between subjects who dropped out and those who did not were found on the variables under study at T1, according to t-test analyses (ps ranging from.486 to.967).

A one-way analysis of variance (ANOVA) was performed to check for potential group differences at T1 between completers of the three groups (see Table 3 for the values). This analysis indicated that there were no significant baseline differences between groups on any of the following variables: subjective happiness, life satisfaction, depression, perceived stress, somatic complaints, and age (ps ranging from.247 to.935). Difference in sex ratios among the three groups was nonsignificant, according to a Fisher's exact test conducted on the number of remaining males and females after dropout among each group (p = .268).

**Table 3:** Means (and Standard Deviations) for Each Scale and Each Group and Significance of Differences Between Time 1 and Time 2 for PER and control group.

	LKM		PER program				Control group			
Scale	Time 1	Time 2	Time 1	Time 2	*t*	*df*	Time 1	Time 2	*t*	*df*
SHS	4.76 (1.44)	5.27 (1.13)	4.51 (1.31)	5.13 (1.16)	−2.94**	26	4.48 (1.58)	4.37 (1.42)	0.41	34
SWLS	4.73 (1.40)	5.21 (1.43)	4.76 (1.09)	5.31 (1.07)	−2.88**	27	4.37 (1.25)	4.55 (1.17)	−1.31	34
BDI	5.5 (5.25)	3.13 (3.34)	7.5 (5.73)	4.32 (4.60)	2.73*	27	6.97 (5.31)	6.26 (5.00)	1.29	34
PSS	2.68 (0.73)	2.34 (0.73)	2.77 (0.71)	2.48 (0.81)	1.97^†^	27	2.76 (0.75)	2.78 (0.74)	−0.22	33
PILL	1.87 (0.37)	1.60 (0.31)	1.85 (0.42)	1.64 (0.40)	3.30**	26	1.80 (0.49)	1.81 (0.53)	−0.18	34

*Note.* ^†^ *=  p*<.1, * = *p*≤.05, ** = *p*≤.*01.* SHS  =  Subjective Happiness Scale; SWLS  =  Satisfaction With Life Scale; BDI  =  Beck Depression Inventory; PSS  =  Perceived Stress Scale; PILL  =  Physical Inventory of Limbic Languidness.

#### Test of our hypothesis

**Figure 1:**
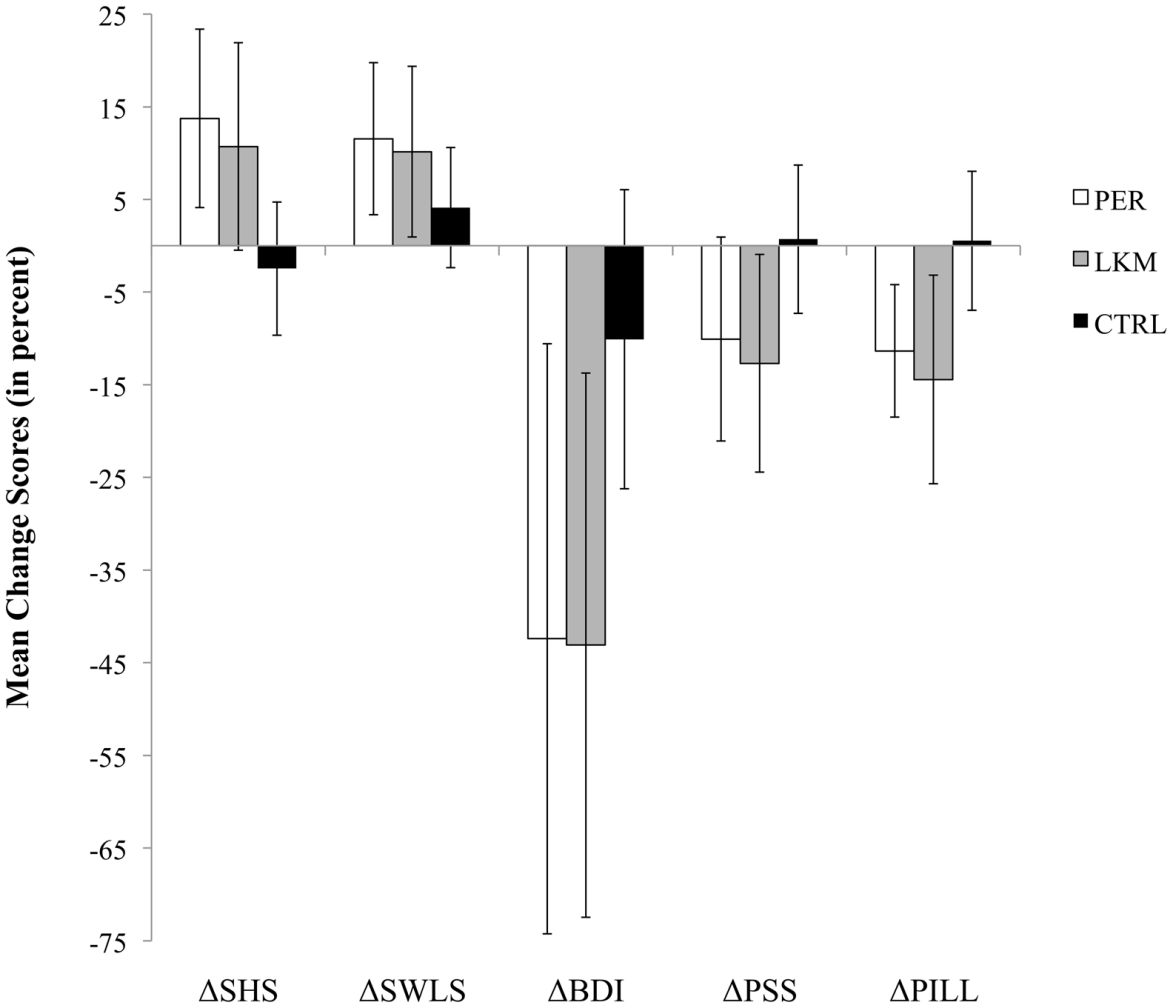
Mean Change Scores (expressed in percent) between Time 1 and Time 2 for the three groups. ΔSHS  =  Mean Change Score on Subjective Happiness Scale; ΔSWLS  =  Mean Change Score on Satisfaction With Life Scale; ΔBDI  =  Mean Change Score on Beck Depression Inventory; ΔPSS  =  Mean Change Score on Perceived Stress Scale; ΔPILL  =  Mean Change Score on Physical Inventory of Limbic Languidness.

In order to assess the evolution of participants' scores within each group, we computed change scores by subtracting T1 scores from T2 scores for each participant on each variable. A graphic depiction of mean change scores (expressed in percent) by group can be found in Figure 1.

Separate one-way ANOVAs with one between-subject factor (three groups: PER, LKM, and control) were then performed on change scores in order to compare the three groups. Analyses showed that the mean difference score for SHS tended to be greater in LKM and PER than in the control group. The effect was only marginally significant (p = 0.08) but the power was low (P = 50%). The mean difference score for BDI tended to also be greater in LKM and PER than in the control group. Again, the effect was only marginally significant (p = 0.09) but the power was low (P = 48%). The mean differences for SWLS and for PSS were not statistically different between groups (ps = .24 and 0.11, respectively), but the power of the analyses was again very low (P = 31% and 44% respectively). Finally, the mean difference score for PILL was statistically different between groups (p =  0.018, P = 73%): the control group differed significantly from LKM (p = 0.044) and marginally from PER (p = 0.065).

Although these results are somewhat informative, the very low powers of the analyses are problematic. *Statistical power* can be interpreted as the probability of finding a significant difference, if it exists, with the present sample sizes and the observed effect sizes. The conclusion that can be drawn from those results is that, for our effects to be statistically significant, we would need a much larger sample size. When power analyses are used to determine the optimal size of a sample before the beginning of a study, standards recommend powers between 80 to 95% [77]. Though the power of our study was far from this standard, this was a pilot study, and as such, we were unable to predict (1) the dropout rate in each group, and therefore the adequate number of participants to initially include in our study, and (2) the average effect size of our intervention, which would have been necessary to compute power analyses before the start of the study.

The high dropout rate in the LKM group dramatically reduced the number of observations and, therefore, considerably reduced the power of analyses including all three groups. Under these conditions, it seems hardly possible to highlight results on the PER group, even though the visual analysis of the change scores (see Fig 1) suggests that some interesting results do exist.

Since the power problems were mainly related to the LKM group, and since our main group of interest was the PER group, we decided to leave the question of LKM efficacy aside, so as to focus on our main question of interest: the efficacy of the PER program. Analyzing those results independently of the LKM seemed to constitute the most relevant option (from both a clinical and statistical point of view) to quantify the effects of the PER intervention, if they exist. In the following pages, we will present the results of analyses comparing the PER and control groups only. The means and standard deviations for each variable at each time point in the PER program and in the control group are shown in Table 3.

Repeated measures ANOVAs were performed on each measure, with group (PER program, control) as a between-subjects factor, and time (T1, T2) as a within-subjects factor. In each case, we were looking for a significant Time x Group interaction, which would indicate a differential change between the two groups. Analyses confirmed a highly significant group interaction for four of the five scales: SHS, F(1, 59) = 5.52, p<.022, η^2^_partial_ = .09; SWLS, F_(1, 60)_ = 4.20, p<.045, η^2^_partial_ = .07; BDI, F_(1, 60)_ = 4.50, p<.038, η^2^_partial_ = .07; and PILL, F_(1,59)_ = 5.86, p<.021, η^2^_partial_ = .09. Results for PSS did not reach significance (F_(1, 59)_ = 2.99, p = .089, η^2^_partial_ = .05).

As depicted in Table 3, the breakdown of these interactions revealed that, unlike participants in the control group, who did not show any change on any variable under study between T1 and T2, participants in the PER group showed a significant increase in subjective happiness (SHS), t (26) = −2.94, p>.007, d = .50, and satisfaction with life (SWLS), *t* (27) = −2.88, p<.008, d = .51. They also showed a significant decrease in depression (BDI), *t* (27) = 2.73, p<.011, d = .61 and physical symptoms (PILL), *t* (26) = 3.30, p<.003, d = .51 and a marginal decrease in perceived stress (PSS), *t* (27) = 1.97, p = .059, d = .38 (see Table 3).

## Discussion

The objective of our study was twofold: (1) to construct a theory- and evidence-based integrative intervention aimed at increasing well-being and (2) to test its effectiveness on participants' mental and physical well-being. Based on the Process Model of Positive Emotion Regulation [28], [29], we developed a 12 hour (6 × 2 hr) integrative training program, bringing together a series of theory-based and empirically-validated techniques. The theoretical framework allowed us to organize these different well-being enhancing techniques and to deliver them in a coherent format. In addition, this framework made it possible to integrate techniques using different underlying processes (or strategies).

In accordance with our hypothesis, our results indicate that, compared to an inactive control group, the PER group showed a significant increase in subjective happiness and satisfaction with life and a significant decrease in depression symptoms and somatic complaints. There was also a marginal decrease in perceived stress. These results confirm that it is possible to enhance an individual's psychological and physical well-being and that the PER program that we developed is a valid intervention to achieve this goal.

Another interesting finding of our study concerns loving-kindness meditation. In our study, the LKM dropout rate (43%) suggests that, although LKM seems to work for those who follow the program until the end, a significant proportion of participants do not adhere to this intervention. It is worth noting that the high dropout rate in the LKM condition is consistent with results reported by Carson et al. [78]. The latter study tested the effects of LKM among chronic pain sufferers and also showed a 42% dropout rate (13 out of 31 subjects) in its LKM group. However, individuals who fully adhered to the intervention until the end of the program benefited from it, as evinced by better psychological adjustment to pain. Several hypotheses can be put forward in order to explain the high dropout in our study. First, participants who enrolled in our program had no precise idea of the type of techniques they were going to learn, unlike in Fredrickson et al.'s [65] well-known LKM study, where participants enrolled in a “meditation program.” It is therefore possible that some of them were surprised by the proposed method and found that it did not suit them, which may have caused them to drop out of the intervention. The majority of the qualitative feedback received from participants who quitted the LKM condition mentions an incongruity between the proposed technique and their personality (e.g. “I am too impatient for this type of exercise,” “Meditation stresses me, I am not comfortable with this type of method,” “If I had known that we were going to meditate, I would not have signed up”). Second, another explanation that may explain the high dropout rate in the LKM group concerns the trainers [79]. As pointed by an anonymous reviewer, the large number of dropouts could have been because some participants did not enjoy the personality of the trainer and/or the type of group dynamic he proposed. In order to reduce the risk of having such a trainer effect, we recruited two LKM trainers. Results indicate that the observed dropout was independent of the trainer identity. For this reason, we believe that this second hypothesis is less plausible.

Overall, our results and previous literature suggest that LKM can be an effective technique to increase the well-being of individuals who adhere to the program. Our results also show that the PER program is an effective alternative that may be more readily accepted by the majority of individuals. Previous LKM results (e.g. [65], [78]) suggest that it may be interesting to include elements of LKM within an integrative intervention such as the PER program, or to recommend this technique to individuals for whom this type of intervention appears to be the most clinically relevant (e.g. people open to meditative practices).

In addition to bringing a theoretical reflection about the processes underlying happiness enhancing techniques, this article aimed to provide practical information to practitioners desiring to use an integrative and theory-driven intervention to boost their clients' well-being. This is why we have described the PER program that we developed based on Quoidbach et al.'s theoretical model [28], [29] in greater detail than usually found in empirical articles. Like one anonymous reviewer, one may wonder whether the underlying theoretical model must be described to the participants. Based on our experience and on participants' qualitative comments at the end of the sessions, we think that it should definitely be explained to them. First, several times during the training, we invite participants to choose the exercises they wish to practice from a selection that we offer (for their weekly homework and long-term exercises after the end of the program). However, the techniques that individuals prefer are not always those that are best for them [69], [80]. Explaining the model to the participants therefore seems important, so as to show them the variety of techniques available and to encourage them to try at least one technique from each type of strategy (or model section) in order to discover new methods to enhance their happiness level. Second, some of our participants reported that the classification of strategies made them aware that they always use the same family of strategies and rarely any of the others. For instance, one participant realized that he often interprets events positively (cognitive level), but that he is systematically incapable of being fully present during the pleasant moments he experiences (attentional level). Other participants reported similar realizations about techniques usable before, during or after an event. One young woman clearly identified that she is able to feel a lot of positive emotions about past events, but that she almost never plans future events in order to get the most out of them. Thus, the model increases participants' awareness of their functioning and provides them with new ways to increase their well-being. We therefore encourage trainers who would like to teach the PER program to present and explain the model to their groups.

In individual therapy, Quoidbach et al.'s model [28], [29] could also be used to identify strategies that a happiness seeker has not been using, and thereby the type of exercise(s) that could be prescribed. However, further research should be conducted to better understand which strategies are the most beneficial for specific profiles of individuals. Pending the development of individually tailored happiness enhancing interventions (see [57], [81] for examples of “tailored interventions” attempts), integrative programs such as the PER program offer the greatest probability of being effective for the widest range of individuals.

## Limitations and Future Research

Although this study offers promising prospects, we acknowledge several limitations that leave ample room for future research to refine our findings. The first limitation concerns the missing data in the LKM condition due to the large dropout rate that we had not expected. The number of missing data in this group strongly decreased the statistical power to a point where we did not have sufficient power to demonstrate effects, even if they existed. Now that we have information about the potential dropout rate in the LKM condition, we could run this study again, but this time calculate the appropriate number of participants to include in the groups in order to have a satisfying remaining power at the end of the study, even if a large portion of participants were to fail to complete the intervention.

The second limitation concerns the timing of the assessments. Our post-intervention measure was carried out only once, four weeks after the end of the intervention. Although most of the literature about happiness enhancing strategies includes T2 assessment right after the end of the last exercise, we chose to collect that information four weeks after the end of our intervention because it seemed methodologically more appropriate to measure the remaining impact of our intervention in individuals' real life. Indeed, right after the intervention, people are still fully impregnated with it, while a few weeks later, they have returned to their life, without any reminder of the techniques proposed within the intervention. Although this timing difference diminishes our ability to compare our results to those obtained with other techniques presented in the literature, the fact that we found results four weeks after the end of our intervention shows that it continues to exert its effects after the end of the intervention. It would have been ideal, of course, to follow participants over a longer period of time to be able to do more sophisticated statistical analyses and to see if the benefits maintain in the long term, after six months or one year. Unfortunately, most of our participants were in their last year at university and left the department a few months after the end of the intervention, which led their student email address to be disabled. We lost contact with most of them right after this T2 measurement.

A third potential limitation of our study concerns the composition of our sample. On the one hand, all the participants in our study were young adults, and as a consequence, our results may not be generalizable to the entire population. However, findings from the meta-analysis carried out by Sin and Lyubomirsky [23] indicate that the benefits of positive psychology interventions tend to increase with age. This observation therefore leads us to speculate that testing our intervention on a “young” sample probably resulted in an underestimation, rather than an overestimation, of its effects. On the other hand, our experimental groups were composed of a very limited number of men (PER, N = 4; LKM, N = 3), and this prevented us from investigating a potential impact of gender on the effectiveness of the intervention. It is therefore risky to affirm that our results can be generalized regardless of participant gender. Nevertheless, existing literature in this field has shown no systematic influence of gender on the effectiveness of happiness enhancing techniques, a fact that increases our confidence in the predictive validity of our results for both sexes.

Using only self-reported measures is a fourth limitation of our study. Although numerous authors insist that using self-evaluation is a sensible logical way to evaluate a subjective experience such as well-being [82], we consider that it is necessary to complement these measures with objective health indicators (such as cortisol level [83]), 360 degree assessments (e.g. ratings of one's happiness by friends, partner; frequent verbal expression of positive emotions, [84]) or experience sampling methods [85] in future studies. This kind of measure should decrease the risk of social desirability effects linked to the use of self-report measures and increase the reliability and objectivity of the findings.

As pointed by an anonymous reviewer, future research would benefit from comparing our PER group to existing integrative interventions such as Seligman et al.'s Group Positive Psychotherapy [27]. This would allow determining more precisely the efficiency of our training compared to other integrative intervention and the added value of the theoretical model that underlies it.

Finally, despite the fact that we present an integrative intervention, we acknowledge that studies that focus on specific techniques are critically important. In addition to enabling the evaluation of the unique effect of a particular technique, these studies are crucial in determining the strategies that are particularly beneficial or harmful for specific sub-populations (e.g. highly depressed people, young adults VS people in the final stage of life, individuals from different cultures) and they help understand the processes underlying the effectiveness of a given technique.

## Conclusion

Embarking on the path towards higher levels of well-being is a demanding journey that requires motivation and considerable effort [82]. In order to help individuals seeking happiness along this path, this study outlines an intervention that offers clinicians and trainers a variety of techniques that target different processes and structures them around a theoretical framework.
